# Mitogenome and phylogenetic analysis of typhlocybine leafhoppers (Hemiptera: Cicadellidae)

**DOI:** 10.1038/s41598-021-89525-5

**Published:** 2021-05-12

**Authors:** Jia Jiang, Xiaoxiao Chen, Can Li, Yuehua Song

**Affiliations:** 1 School of Karst Science, State Key Laboratory Cultivation Base for Guizhou Karst Mountain Ecology Environment of China, Guizhou Normal University, Guiyang, 550001 Guizhou China; 2 Guizhou Provincial Key Laboratory for Rare Animal and Economic Insect of the Mountainous Region/Guizhou Provincial Engineering Research Center for Biological Resources Protection and Efficient Utilization of the Mountainous Region, Guiyang University, Guiyang, 550005 Guizhou China

**Keywords:** Evolution, Genetics, Molecular biology, Systems biology

## Abstract

Mitogenomes have been widely used to estimate phylogenetic relationships among insects and provide data useful for augmenting traditional morphological characters in delimiting species. Here, complete mitogenome sequences of two closely related typhlocybine leafhoppers, *Cassianeura* *cassiae* (Ahmed, 1970) and *C*. *bimaculata* Dworakowska, 1984, were obtained and found to be 15,423 bp and 14,597 bp in length, respectively. The gene order was found to be similar to other published leafhopper mitogenomes, but the control region of *C.* *bimaculata* is the shortest among known leafhoppers and lacks tandem repeats. Phylogenetic analysis of 13 protein-coding genes (PCGs), the first and second codons of 13 PCGs, 13 PCGs and two rRNAs formed three well-supported tree topologies. The topologies of phylogenetic trees inferred from three datasets were almost identical, which was consistent with previous molecular phylogenies of this group. Comparative morphological study of the ovipositors revealed several characters potentially useful for diagnosing genera and resolving their phylogenetic relationships. Phylogenetic analysis of these and other morphological characters yielded a tree that is mostly consistent with the tree obtained from analysis of mitogenome sequences. In both molecular and morphological phylogenenies, Typhlocybini and Zyginellini clustered into one clade, but neither was recovered as monophyletic.

## Introduction

Typhlocybinae (Hemiptera, Auchenorrhyncha, Cicadellidae) is the second-largest leafhopper subfamily after Deltocephalinae, and comprises 510 genera, and 4929 species, widely distributed in the six major zoogeographic regions of the world^1^. Unlike most other Cicadellidae, members of this subfamily feed directly from plant cells and a strong host specificity^2,3^. Many are also important agricultural pests.

Traditionally, the tribes of Typhlocybinae are distinguished based on the fore and hind wing venation, but the tribal classification of Typhlocybinae has been unstable for a long time. Oman et al. proposed that Typhlocybinae consist of ten effective tribes^4^, and Young believes four tribes^5^. Currently six tribes are widely recognized by Dworakowska: Alebrini, Dikraneurini, Empoascini, Erythroneurini, Typhlocybini and Zyginellini. He divided Zyginellini into a separate tribe based on the species whose hind wing submarginal vein apparently connected directly to CuA rather than being joined by a crossvein^6^. Although Zyginellini has a unique hindwing venation, most genera in this tribe strongly resemble many Typhlocybini, especially in the structure of the male genital capsule and aedeagus^7^. Thus, more data should be used to further study their phylogenetic relationship.

The insect mitogenome is usually a covalently closed circular double-stranded DNA molecule, usually ~ 16 kb in size, containing 13 protein-coding genes (PCGs), 22 transfer RNA (tRNA) genes, and two ribosomal RNA (rRNA) genes and a non-coding region (A + T-rich region)^8^. Owing to its simple structure, small size, multiple copies, maternal inheritance, lack of recombination, and rapid evolution^9,10^, it is widely used to estimate phylogenetic relationships at various taxonomic levels and to confirm morphological homologies^11^.

In recent years, advances in sequencing technology and the application of universal primers for mitochondrial genes^12,13^ have facilitated acquisition of large numbers of insect mitochondrial genomes. So far, however, complete mitogenome data for only 15 species of Typhlocybinae have been deposited in the National Center for Biotechnology Information (NCBI), including four species of Erythroneurini, three species of Typhlocybini, four species of Zyginellini, and four species of Empoascini. These data are therefore far from sufficient to facilitate detailed comparative and phylogenetic research on Typhlocybinae. In order to supplement of available mitogenome data of Typhlocybinae and exploration of its internal phylogenetic relationship, two species of the genus *Cassianeura* of the Erythroneurini were sequenced and annotated in this study (Table 1). The structure and composition of mitochondrial genomes of two of the three species of *Cassianeura* were analyzed and compared. Specimens of the two species were recently collected by us from the *Cassia* tree in Pattaya, Chunburi Province, Thailand^14^.

**Table 1:** Information on samples.

subFamily	Species	Length(bp)	GenBank No	Sampling time	Collecting sites	Collector
Typhlocybinae	*Cassianeura* *cassiae* (Ahmed, 1970)	15,423	MT985380	26 I 2019	Thailand	Can Li
	*Cassianeura* *bimaculata* Dworakowska, 1984	14,597	MT985381	26 I 2019	Thailand	Can Li

## Materials and methods

### Sample collection and DNA extraction

The specimen information for leafhoppers collected in this study are shown in Table 1, and the data downloaded from GenBank are shown in Table 2. The specimens were immersed in absolute ethanol and stored in a refrigerator at − 20 °C. After morphological identification, the prepared voucher specimens with male genitalia and female genitalia were deposited in the insect specimen room of Guizhou Normal University.

**Table 2:** Sequence information of 17 species of Typhlocybinae and one outgroup downloaded from GenBank.

tribe	Species	Length (bp)	GenBank no.	References
Erythroneurini	*Mitjaevia* *protuberanta* Song, Li & Xiong, 2011	15,472	NC_047465.1	^24^
	*Empoascanara* *dwalata* Dworakowska, 1971	15,271	MT350235.1	^25^
	*Empoascanara* *sipra* Dworakowska, 1980	14,827	NC_048516.1	^26^
	*Illinigina* sp.	14,803	KY039129.1	^27^
Typhlocybini	*Eupteryx* *minuscula* Lindberg, 1929	16,945	MN910279.1	^28^
	*Bolanusoides* *shaanxiensis* Huang & Zhang, 2005	15,274	MN661136.1	^29^
	*Typhlocyba* sp.	15,233	KY039138.1	^27^
Zyginellini	*Limassolla* *lingchuanensis* Chou & Zhang, 1985	15,716	NC_046037.1	^30^
	*Zyginella* *minuta* (Yang, 1965)	15,544	MT488436.1	^31^
	*Paraahimia* *luodianensis* Yuan & Song, 2019	16,497	NC_047464.1	^32^
	*Parathailocyba* *orla* (Dworakowska, 1977)	15,382	MN894531.1	^33^
Empoascini	*Empoasca* *onukii* Matsuda, 1952	15,167	NC_037210.1	^34^
	*Empoasca* *vitis* (Göthe, 1875)	15,154	NC_024838.1	^35^
	*Empoasca* sp.	15,152	MK211224.1	^36^
	*Ghauriana* *sinensis* Qin & Zhang, 2011	15,491	MN699874.1	^37^
Outgroup (Cercopidae)	*Phymatostetha* *huangshanensis* Ouchi, 1943	17,785	NC_039157.1	^38^
	*Callitettix* *braconoides* (Walker, 1858)	15,637	NC_025497.1	^39^

### Genome sequencing, assembly, and annotation

Mitogenomes were obtained by PCR amplification and sequencing. The PCR reaction was performed with LA-Taq polymerase. The cycling conditions comprised a predenaturation step for 2 min at 94 °C, followed by 35 cycles of denaturation at 94 °C for 30 s, annealing at 55 °C for 30 s, elongation at 72 °C for 1 min/kb, and then the final extension for 10 min at 72 °C. PCR products were then directly sequenced. After quality-proofing of the obtained DNA fragments, DNAStar^15^ was used to manually assemble the complete mt genome sequence, and homology search was performed through the Blast function in NCBI to verify whether the amplified sequence matched the target gene regions^16,17^.

The positions and secondary structures of 22 tRNA genes were determined using tRNAscan SE version 1.21^18^ and ARWEN version 1.2^19^. The base composition of each gene, relative synonymous codon usage (RSCU), and A + T content values were analyzed with MEGA 6.06^20^. Tandem repeat sequences of the control area were identified by the online search tool Tandem Repeats Finder^21^. Strand asymmetry was calculated through the formula: AT-skew = [A − T]/[A + T] and GC-skew = [G − C]/[G + C]^22^. DnaSP 5.0 software^23^ was used to estimate the ratio of the non-synonymous (Ka) to the synonymous substitution rate (Ks) of 13 PCGs and calculate the nucleotide diversity (Pi) of 13 PCGs which from 23 species of Typhlocybinae. The sliding window analysis was set to sliding window: 200 bp, step size: 20 bp.

### Phylogenetic analysis

A molecular phylogenetic analysis was performed based on mitogenomes of 17 species in Typhlocybinae, and two species from Cercopidae was selected as an outgroup (Tables 1 and 2). To overcome of base compositional heterogeneity and explore the effect of method choice on phylogenetic reconstruction, three datasets were assembled: (1) a concatenated nucleotide sequence alignment of the first and second codons of 13 PCGs (PCG12); (2) a concatenated nucleotide sequence alignment of 13 PCGs (PCG123); (3) a concatenated nucleotide sequence alignment of the first and second codons of 13 PCGs and two rRNAs (PCR12R). The Gblocks Server online platform was used to eliminate poorly aligned positions and divergent areas of the DNA protein alignment and check and correct all the alignments in MEGA 6.06^40^. Phylogenetic trees were estimated using the Bayesian Inference (BI) method and the Maximum Likelihood (ML) method. The BI analysis was performed using MrBayes 3.2.7^41^. BI selected GTR + I + G as the optimal model, running 10 million generations twice, sampling once every 1000 generations, with a burn-in of 25%, and the remaining trees used to generate a consensus tree and calculate the probability of each branch of the posterior probability (PP). The ML analysis was conducted using IQ-TREE^42^ under an ML + rapid bootstrap (BS) algorithm with 10,000 replicates used to calculate bootstrap scores for each node (BP).

### Photographing and illustration of female genitalia

An Olympus SZX16 dissecting microscope was used for specimen study and an Olympus BX53 stereoscopic microscopes for drawing of the dissected female genitalia. Morphological terminology used in this study follows Dietrich & Rakitov^43^. All specimens examined were deposited in the collection of the School of Karst Science, Guizhou Normal University, China (GZNU). Photographs were taken by using a KEYENCE VHX-5000 digital microscope.

### Morphology-based phylogenetic analysis

63 morphological features of 14 species of Typhlocybinae and 1 species of Dikraneura (outgroup) were selected to establish a morphological phylogenetic tree, of which 8 new characteristics from the female valvulae I, II and III of the ovipositor were choosen for the differences of the female genitalia of leafhoppers (Table S3). The remaining 55 are from Dietrich & Dmitriev ^44^, representing characteristics that show obvious differences among Typhlocybinae. The character states of the female genitalia are unordered and assigned state numbers "0,1,2,3,…". For the remaining characters, "0" represents the hypothesized ancestral state, and "1" represents the derived state. For multi-state characters, states are arranged in order from ancestral to derived. A data matrix compiled based on the above codes was then analyzed using the traditional search method in TNT.

## Results and discussion

### Mitochondrial genomic structure and composition

**Figure 1:**
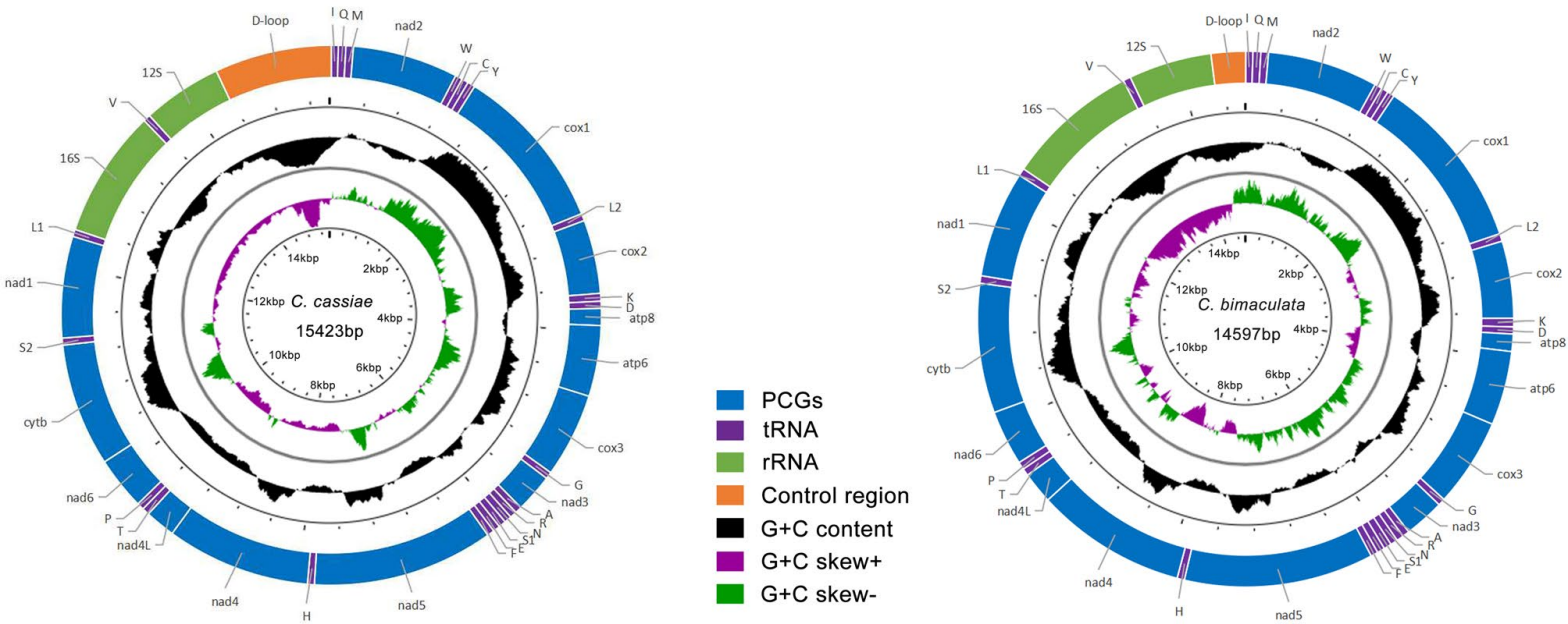
The organization of the mitogenomes of *C.* *cassiae* and *C.* *bimaculata.*

Preivously known mitogenomes of Typhlocybinae range from 14,803 bp to 16,945 bp^27,28^ (Table 2). The complete mitogenomes of *C.* *cassiae* and *C.* *bimaculata* are 15,423 bp and 14,597 bp in size, respectively (Fig. 1, Table 1). The smaller size of the complete mitogenome of *C.* *bimaculata* is mainly due to the difference in the length of the A + T-rich (control) region. The mitogenomes of *C.* *cassiae* and *C.* *bimaculata* contain the usual 13PCGs, 22 tRNA genes, two rRNA genes and non-coding regions found in other insects, no gene rearrangements are present. Twenty-three genes encode in the minority strand (H-strand) while the others encode in the majority strand (J-strand) (Table S1).

The mitogenome of *C.* *cassiae* has 12 intergenic spacers, 47 bp in total, ranging in length from 1 to 10 bp. The longest intergenic spacer is between *trnA* and *trnR*; 14 gene overlap regions are present, ranging in length from 1 to 8 bp, with the longest between *trnW* and *trnC* (Table S1). The mitogenome of *C.* *bimaculata* has 10 intergenic spacers of 41 bp in total, and the length also varies from 1 to 10 bp. The longest intergenic spacer is between *trnQ* and *trnM*; 14 gene overlap regions are also present in this species, ranging in length from 1 to 8 bp, with the longest gene in the same position as in *C.* *cassiae* (Table S1). Such gene structure is common among leafhoppers^27,45^.

The A + T content of *C.* *cassiae* and *C.* *bimaculata* (Table 3) is 78.4–93.6% and 73.3–87.8%, respectively. The highest A + T content of both appears in the control region (CR), the lowest value of *C.* *bimaculata* appears in the 1st codon position while in *C.* *cassiae* it occurs in the N-strand. All genes of the two species mostly showed positive AT-skew and negative GC-skew. The nucleotide composition of the J-strand of *C.* *cassiae* and *C.* *bimaculata* show relatively high A + T content, 86.2% and 79.3%, respectively, consistent with most other metazoan species^46^. Such mitogenome structure is common in Arthropoda^47,48^. However, the N-strand of *C.* *cassiae* and *C.* *bimaculata* show negative AT-skew and negative GC-skew.

**Table 3:** Nucleotide composition of the *C.* *cassiae* (C) and *C.* *bimaculata* (B) mitogenomes.

Feature	A%		C%		G%		T%		A + T%		AT-skew		GC-skew		Length(bp)	
	C	B	C	B	C	B	C	B	C	B	C	B	C	B	C	B
Whole	42.8	39.9	10.4	11.8	8.5	10.6	38.3	38.7	81.1	78.6	0.055	0.015	− 0.101	− 0.054	15,423	14,597
PCGs	42.2	38.5	10.9	12.3	9.4	11.4	37.5	37.8	79.7	76.3	0.058	0.009	− 0.074	− 0.038	10,966	10,967
1st codon position	44.5	40.5	9.6	13.3	8.7	13.5	37.1	32.8	81.6	73.3	0.091	0.105	− 0.049	0.007	3656	3656
2nd codon position	41.4	35.6	10.5	14.3	9.8	11.3	38.2	38.8	79.6	74.4	0.040	− 0.043	− 0.034	-0.117	3655	3656
3rd codon position	40.6	39.5	12.6	9.2	9.8	9.3	37.9	42.9	78.5	82.4	0.034	− 0.041	− 0.125	0.005	3655	3655
tRNA	41.0	40.0	11.4	11.0	9.6	10.5	38.1	38.5	79.1	78.5	0.037	0.019	− 0.086	− 0.023	1453	1433
*16S*	47.9	47.7	10.0	10.4	6.2	6.1	36.0	35.8	83.9	83.5	0.142	0.143	− 0.235	− 0.261	1184	1168
*12S*	48.7	46.2	10.2	11.8	6.0	6.8	35.1	35.2	83.8	81.4	0.162	0.153	− 0.259	− 0.269	733	731
CR	41.7	42.4	4.1	5.0	2.3	7.3	51.9	45.4	93.6	87.8	− 0.109	− 0.034	− 0.281	0.187	1078	302
N	36.6	33.3	11.0	12.3	10.7	12.0	41.8	42.4	78.4	75.7	− 0.066	− 0.120	− 0.02	− 0.01	7680	7664
J	50.1	47.3	10.7	11.6	7.1	9.0	36.1	32.0	86.2	79.3	0.162	0.193	− 0.202	− 0.126	6656	6635

### Protein-coding genes and codon usage

As in most leafhoppers, only four genes (*nd5*, *nd4*, *nd4L*, and *nd1*) of the 13 PCGs of *C.* *cassiae* and *C.* *bimaculata* are encoded on the N-strand, while other genes are encoded on the J-strand (Table S1). Except for *atp8* and *nad5* that use TTG as the start codon, the remaining 13 PCGs start codons all follow the ATN rule. Among the 13 PCGs, standard stop codons (TAA and TAG) and an incomplete stop codon (T) are used. The *cox2* of both species are terminated by T, the *nad5* gene of *C.* *bimaculata* is also terminated by T, but the *nad5* gene of *C.* *cassiae* is terminated by TAA. Most of the start and stop codons of the two species are the same; only the four genes *nad2*, *atp8*, *nad3*, and *nad5* are different. Such subtle differences are common among Cicadellidae^45,49^.

**Figure 2:**
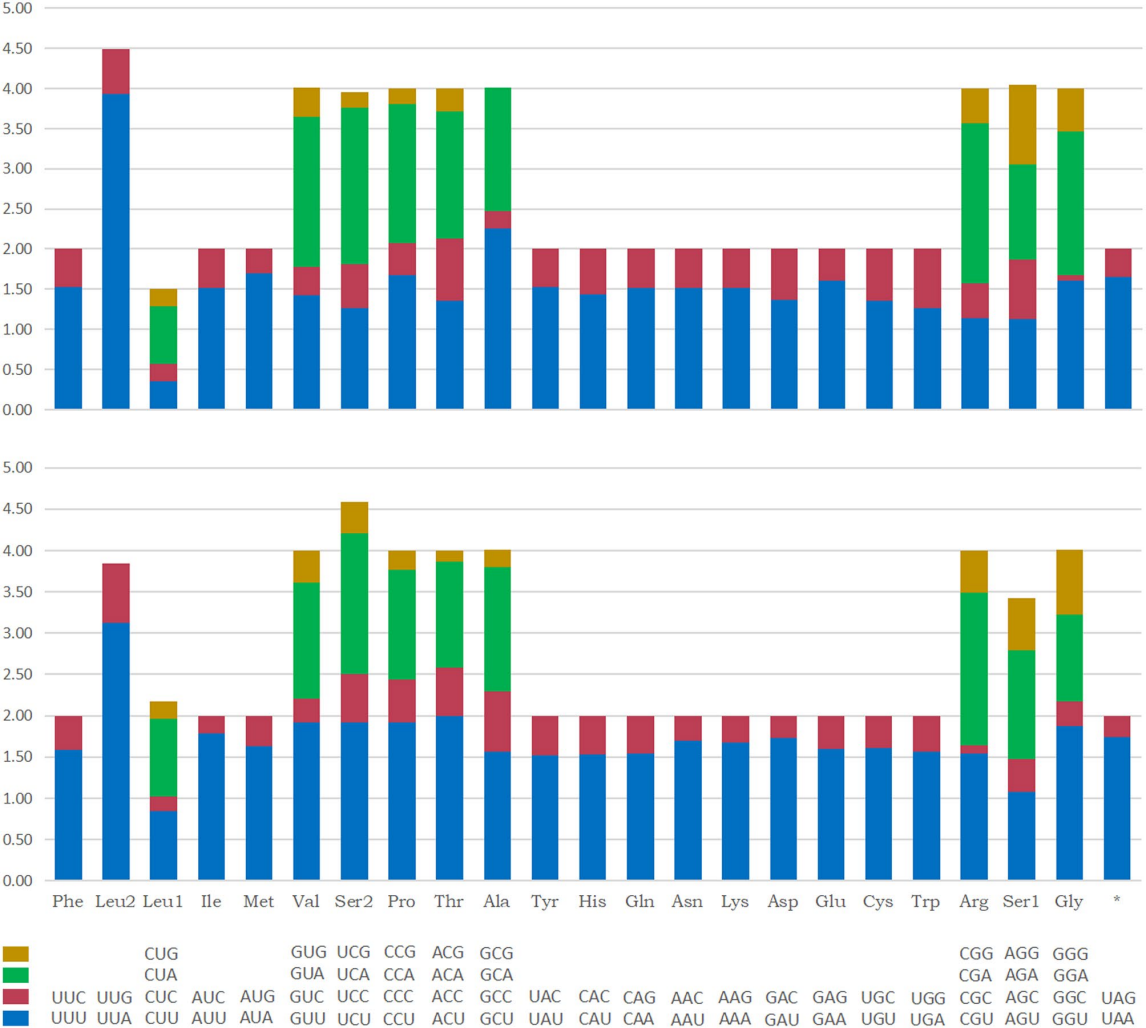
Relative synonymous codon usage (RSCU) of mitogenomes for *C.* *cassiae* (top) and *C.* *bimaculata* (bottom).

The relative synonymous codon usage (RSCU) for the 13 PCGs is summarized in Table S2 and Fig. 2. Both *C.* *cassiae* and *C.* *bimaculata* genes encode a total of 3,655 amino acids. In the two mitochondrial genomes, the frequency of the codon UUA (Leu2) is much higher than other codons, and the RSCU for the two species is 3.93 and 3.12, respectively. The codon GCG (Ala) has not been found in *C.* *cassiae*, and absence of this codon has been observed in previous studies. For example, *Ricania* *speculum* (Fulgoroidea: Ricaniidae) lacks Thr (ACG), and *Aphaena* *discolor* *nigrotibiata* (Fulgoridae) lacks CCG (Pro), GCG (Ala) and ACG (Thr)^50,51^. In addition, both species showed greater codon bias.

### Transfer and ribosomal RNA genes

The total tRNA lengths of *C.* *cassiae* and *C.* *bimaculata* are 1453 bp and 1433 bp, respectively, of which 14 tRNAs are on the N-strand, and the rest are on the J-strand. The 22 tRNAs of the two species are between 62 and 72 bp in length. The *trnK* are longest of the two species, and the shortest tRNA of *C.* *cassiae* is *trnC* while *C.* *bimaculata* has many, including *trnC*, *trnY*, *trnD*, *trnG*, *trnA*, and *trnR* (Table S1). The secondary structure of 22 tRNAs of the two species are shown in Fig. S1.

Secondary structures of tRNAs of *C.* *cassiae* and *C.* *bimaculata* resemble those of other leafhoppers, except that the dihydrouridine (DHU) arm of *tRNA-S1* forms a simple loop, the remaining 21 tRNAs exhibit a typical clover-leaf secondary structure^45,52,53^. 17 and 16 weak G-U base pairs are found in the tRNAs of *C.* *cassiae* and *C.* *bimaculata*, respectively. In addition, there are differences in the number of base pairs on the Anticodon arm of Arg (R), Asp (D), and Met (M) of the two species. This difference is not common in two species of the same genus^45,49^.

The two highly conserved rRNA genes of *C.* *cassiae* and *C.* *bimaculata* are encoded on the short-chain (J-strand). *16S* are located between *trnL2* and *trnV*, with lengths of 1184 bp and 1168 pb, respectively, and *12S* are located after *trnV*, with lengths of 733 bp and 731 bp, respectively (Table S1). Both *16S* and *12S* have positive AT-skew and negative GC-skew (Table 3).

### Control region

**Figure 3:**
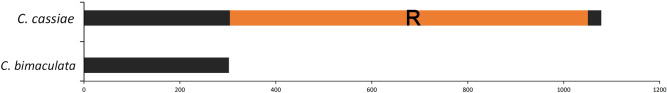
Structural organization of the Control region of *C.* *cassiae* and *C.* *bimaculata.* R: repeat unit.

The control region, also known as the A + T region, is the initiation region of mitochondrial DNA replication and the largest non-coding region in the metazoan mitogenome; due to variation in the length of tandem repeat units (TRs) and the difference in copy number, the control region has much greater size variation than other regions of the mitotic genome^48,54,55^. The lengths of the control regions in the mitotic genomes of *C.* *cassiae* and *C.* *bimaculata* are 1078 bp and 302 bp, respectively, and the A + T% are 93.6% and 87.8%, respectively (Table 3). The control region of *C.* *cassiae* has TRs with 746 bp and 289 bp repeating units (Fig. 3). The length of the control region of *C.* *bimaculata* is the smallest known so far in the Typhlocybinae and lacks TRs. So far, no obvious patterns have been found in structural changes in the control region of different leafhopper species.

### Molecular phylogeny

**Figure 4:**
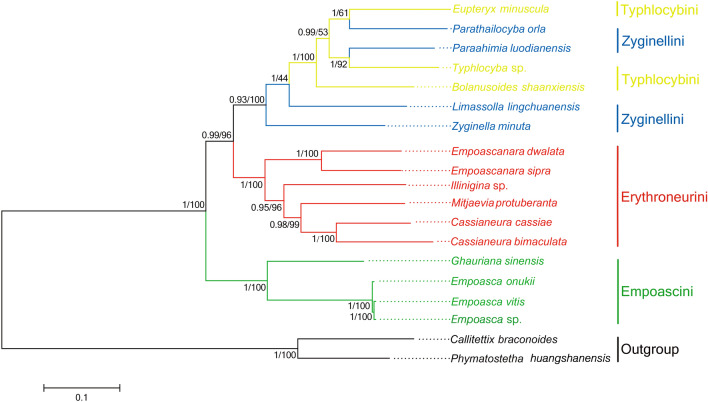
Phylogenetic tree from Typhlocybinae based on nucleotide sequence of 13 PCGs. Numbers above the nodes refer to the posterior probability (left) of Bayesian (BI) analyses and bootstrap proportion (right) of maximum likelihood (ML) analyses.

The phylogeny of Typhlocybinae was established using three concatenated nucleotide datasets of 19 species (17 Typhlocybinae and two outgroups). The topologies of phylogenetic trees inferred from three datasets were almost identical (Figs. 4, S2, S3). The BI and ML analysis produced a consistent tree topology comprising ((Zyginellini + Typhlocybini) + Erythroneurini) + Empoascini (Fig. 4), with the two *Cassianeura* species forming a sister group with *Mitjaevia* *protuberanta*. However, some species of Zyginellini were included in Typhlocybini with low branch support (PCG123: PP = 0.99, BP = 53; PP = 1, BP = 44), findings which add to the controversy regarding the monophyly of these tribes^56^. Zyginellini was erected by Dworakowska, 1977 based on the hind wing vein CuA connected to vein MP compared to their separation in Typhlocybini^57^. This distinction has been accepted for many years, e.g., Zhang^58^ but more systematic analyses are clearly needed before conclusions can be made on the validity of the tribe.

### Female genitalia

**Figure 5:**
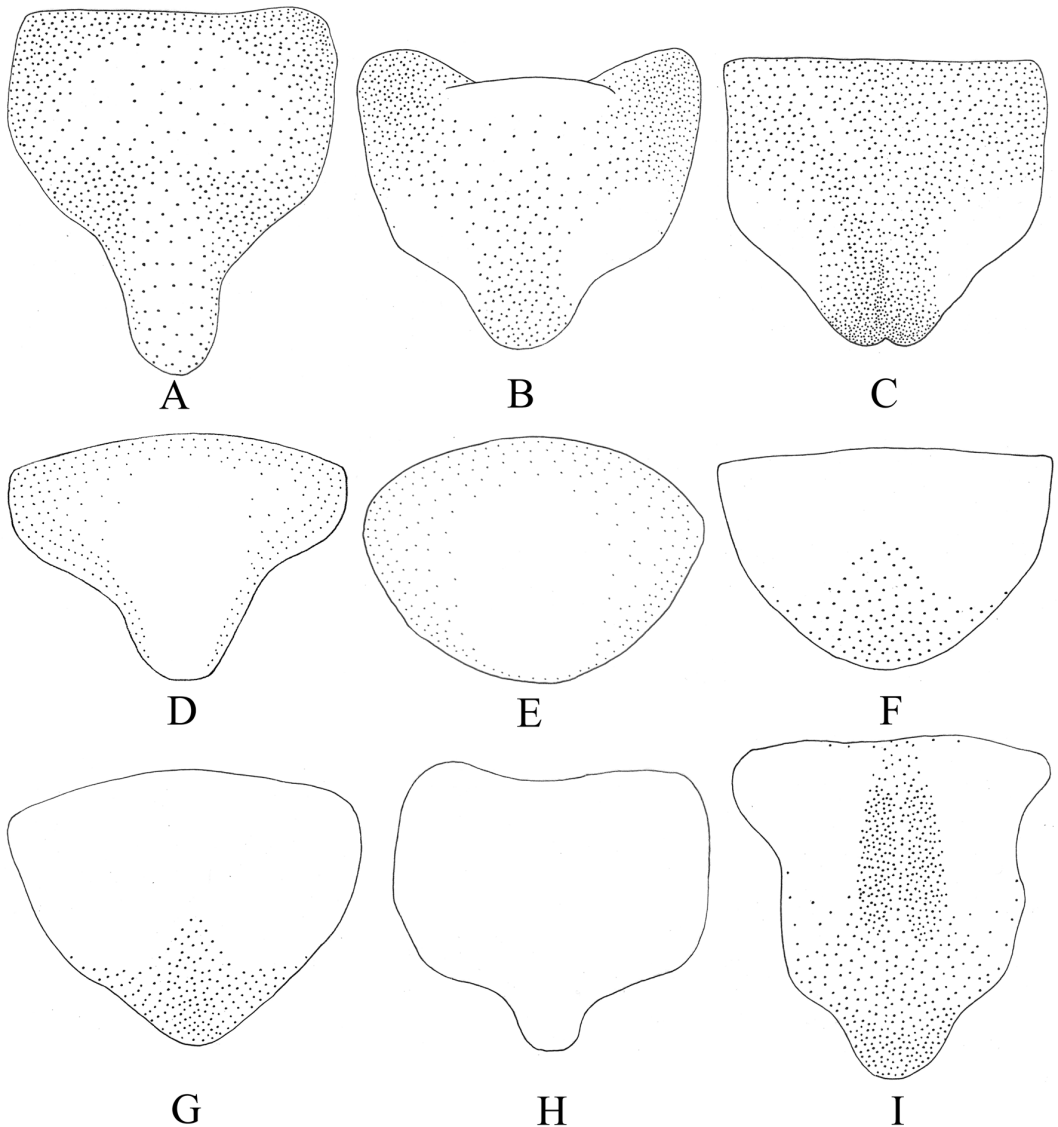
Female sternite VII: **(A)** *Mitjaevia* *protuberanta*. **(B)** *Empoascanara* *sipra*. **(C)** *Empoascanara* *dwalata*. **(D)** *Cassianeura* *cassiae*. **(E)** *Cassianeura* *bimaculata*. **(F)** *Eupteryx* *minuscula*. **(G)** *Eupteryx* *gracilivramus*. **(H)** *Limassolla* *lingchuanensis*. **(I)** *Zyginella* *minuta*.

Previous taxonomic studies have shown that different leafhopper species may vary in the shape of female abdominal sternite VII^43,59–61^. Female sternite VII of nine species in subfamily Typhlocybinae were compared for this study (Fig. 5). All have a convex posterior margin narrower than the base, and a transverse or slightly convex base; except for *E.* *sipra*, which has the base distinctly emarginate. Sternite VII of different species differs in coloration, length, and in the shape of the posterior margin. *M.* *protuberanta* has a relatively long female sternite VII, which covers about half of the ovipositor, and isstrongly produced posteriorly; the female sternite VII of *E.* *sipra* and *C.* *cassiae* conceal the basal third of the ovipositor, with a shorter, rounded medial lobe; the female sternite VII of *E.* *dwalata* has the posterior margin slightly concave medially; the posterior margins of *C.* *bimaculata*, *E.* *minuscula* and *E.* *gracilivramus* are less produced than in other species, being broadly and shallowly rounded posteriorly; the female sternite VII of *L.* *lingchuanensis* is transparent; the female sternite VII of *Z.* *minuta* covers most of the ovipositor, with two longitudinal dark oval areas in the middle.

**Figure 6:**
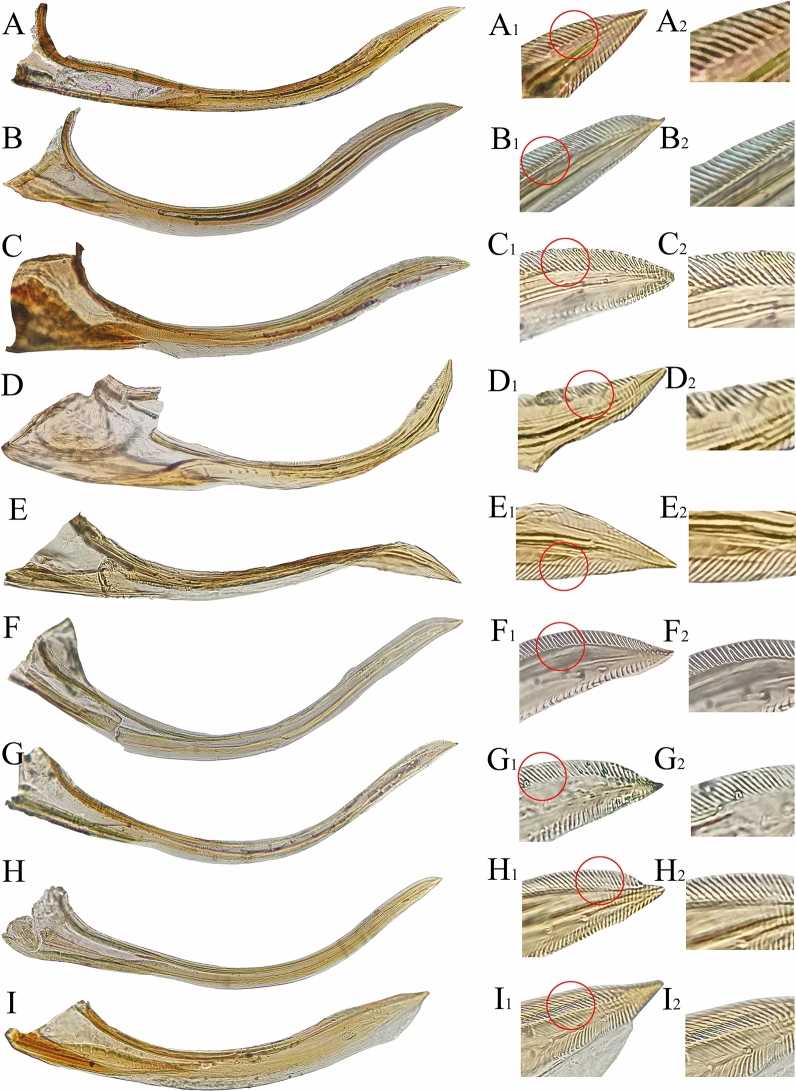
Female valvula I: **(A–I)**: **(A)** *Mitjaevia* *protuberanta*. **(B)** *Empoascanara* *sipra*. **(C)** *Empoascanara* *dwalata*. **(D)** *Cassianeura* *cassiae*. **(E)** *Cassianeura* *bimaculata*. **(F)** *Eupteryx* *minuscula*. **(G)** *Eupteryx* *gracilivramus*. **(H)** *Limassolla* *lingchuanensis*. **(I)** *Zyginella* *minuta*. **(A**_**1**_**–I**_**1**_**)**: Enlarged end. **(A**_**2**_**–I**_**2**_**)**: Local enlargement.

The female valvulae I of the nine species are shown in Fig. 6. The paired valvulae I are connected basally by membranes. Each valvula I is wide at the base and tapers toward the apex, with imbricate sculpture dorsally and ventroapically. Besides the valvula I of *Z.* *minuta,* which is relatively wide, other species are elongated. In previous studies, most typhlocybine leafhoppers have been shown to have long and slim valvulae I^59,60^, with wide valvulae I uncommon. However, Most Deltocephalinae and Cicadellinae leafhoppers have wider female valvulae I^43,61,62^. In addition, the end of female valvula I of *E.* *dwalata* is slightly blunt, while the remaining species are acute.

**Figure 7:**
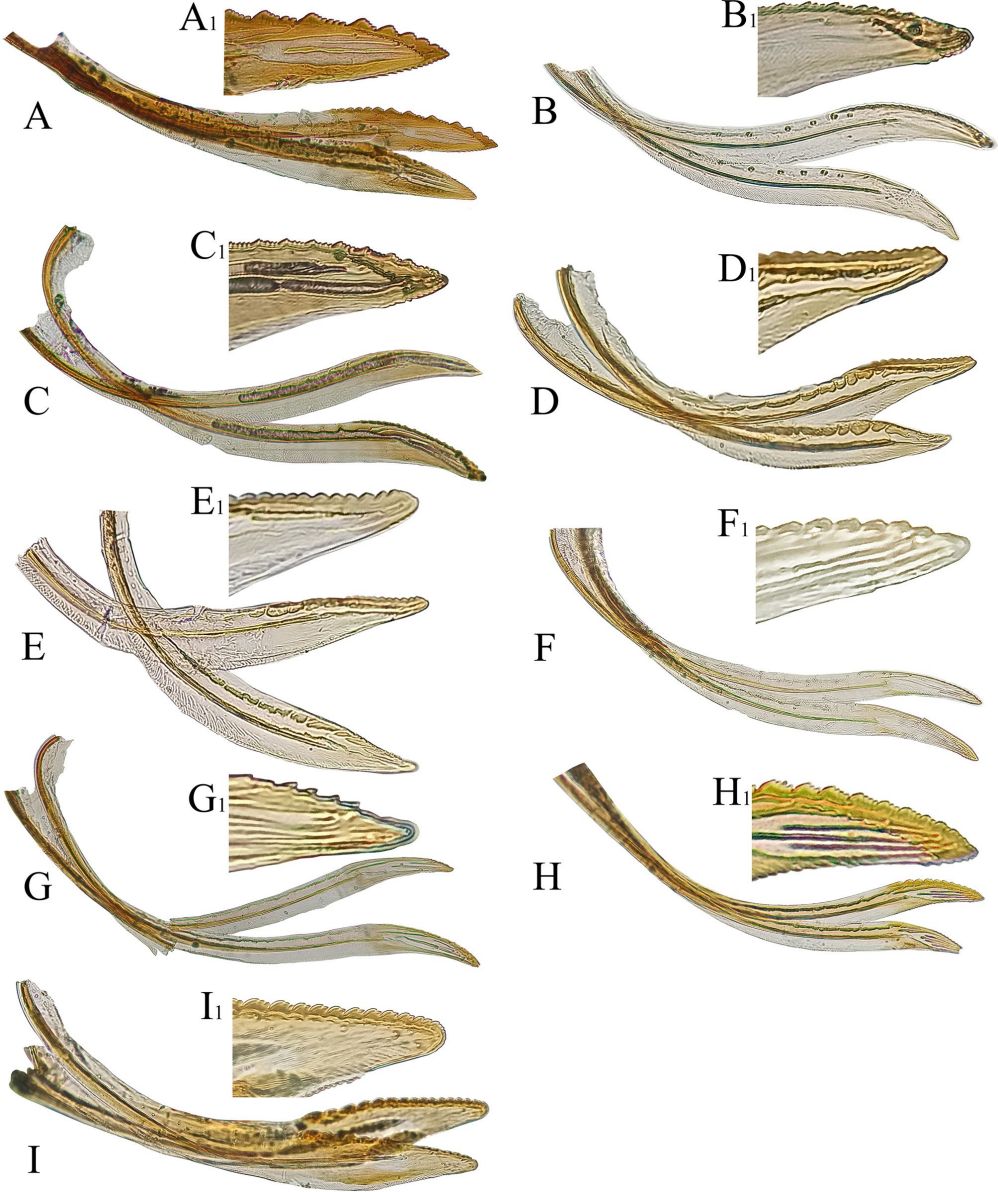
Female valvula II: **(A–I)**: **(A)** *Mitjaevia* *protuberanta*. **(B)** *Empoascanara* *sipra*. **(C)** *Empoascanara* *dwalata*. **(D)** *Cassianeura* *cassiae*. **(E)** *Cassianeura* *bimaculata*. **(F)** *Eupteryx* *minuscula*. **(G)** *Eupteryx* *gracilivramus*. **(H)** *Limassolla* *lingchuanensis*. **(I)** *Zyginella* *minuta*. **(A**_**1**_**–I**_**1**_**)**: Enlarged end.

The valvulae II are divided into two branches and in Typhlocybinae the two branches are slightly different in shape, a unique characteristic of this subfamily^43^. It is unique trait that appears the asymmetry of the right and left valvulae for Typhlocybinae in Cicadellidae. The female valvulae II of the 9 species of leafhoppers examined here are shown in Fig. 7. In lateral view, valvulae II are narrow at the base, distinctly expanded in the middle and then gradually narrowed towards the apex, which is blunt. Valvulae II of *M.* *protuberanta* and *L.* *lingchuanensis* have coarse serrations on the dorsal margin apically, the area between each larger tooth has many small serrations, and the ventral preapical tooth is distinct. *Z.* *minuta* also has some stout serrations on the dorsal margin and the part between each larger tooth also has numerous small serrations. However, compared with *M.* *protuberanta* and *L.* *lingchuanensis*, *Z.* *minuta* has more blunt serrations on the dorsal side, and the ventral apical teeth are absent. The valvulae II of *E.* *sipra* and *E.* *dwalata* have serrations of varying sizes on the dorsal side and the ventral apical tooth is present. *C.* *cassiae* and *C.* *bimaculata* have almost uniform serrations on the dorsal margin apically and ventral margin smooth. The valvulae II of *E.* *minuscula* and *E.* *gracilivramus* have slanted large serrations at the apex dorsally, but *E.* *gracilivramus* has sharper serrations on the dorsal side. The valvulae II of most leafhoppers has an obvious saw-like structure, which is used to cut the leaf epidermis during oviposition^63^.

**Figure 8:**
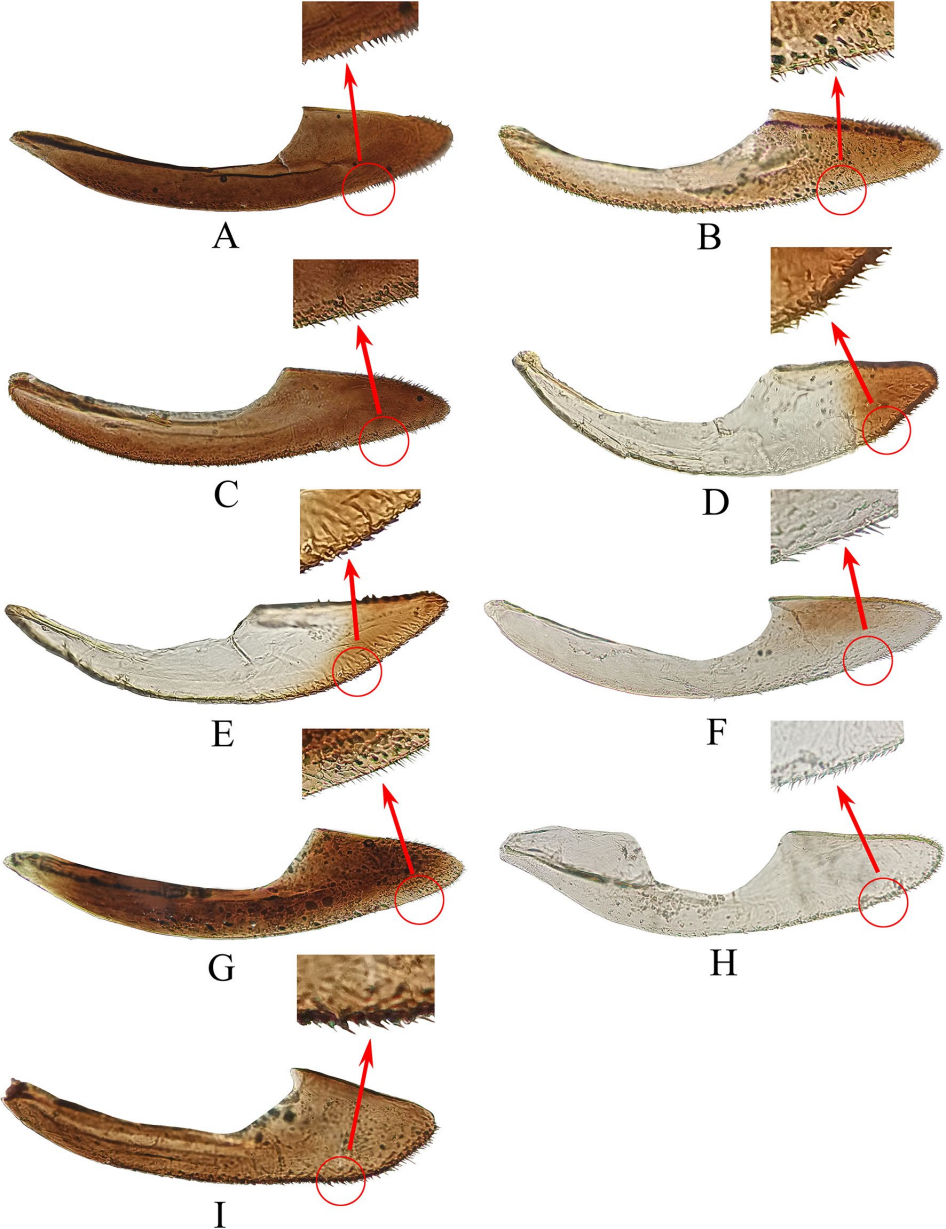
Female valvula III: **(A–I)**: **(A)** *Mitjaevia* *protuberanta*. **(B)** *Empoascanara* *sipra*. **(C)** *Empoascanara* *dwalata*. **(D)** *Cassianeura* *cassiae*. **(E)** *Cassianeura* *bimaculata*. **(F)** *Eupteryx* *minuscula*. **(G)** *Eupteryx* *gracilivramus*. **(H)** *Limassolla* *lingchuanensis*. **(I)** *Zyginella* *minuta*.

The female valvula III of 9 species of leafhoppers is shown in Fig. 8. Valvulae III form a sheath partially enclosing valvulae I and II. Valvula III has its base connected with valvula I and is shaped like a small boat, with some irregularly arranged small setae on the ventral surface. *M.* *protuberanta*, *E.* *sipra*, *E.* *dwalata*, *E.* *gracilivramus*, and *Z.* *minuta* have many slender and dense setae on surface of valvula III, while the other studied leafhoppers have relatively few setae. The color of valvula III of different leafhopper species is quite different, mostly similar to the color of sternite VII^43,61^.

**Figure 9:**
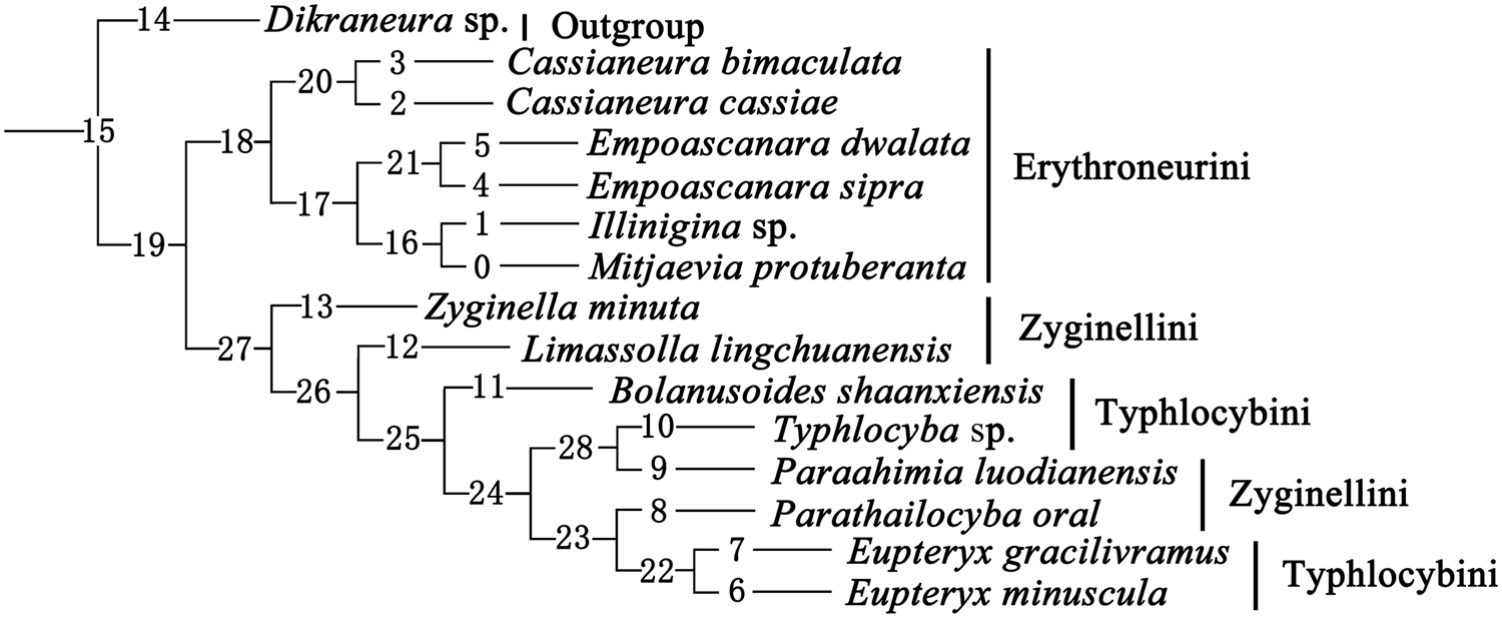
Phylogenetic tree established by traditional search methods based on 63 morphological traits. (Outgroup: *Dikraneura* sp.).

In our comparative study of the ovipositors of 9 existing species, we found that the female valvulae I, II, and III of species in the same genus and tribe are similar to each other. Combining the characteristics of body morphology and male genitalia, a morphological phylogenetic tree was obtained (Tables S3, S4, Fig. 9). The internal relationship of the morphological phylogenetic tree is: (Zyginellini + Typhlocybini) + Erythroneurini. Comparing with the molecular phylogenetic tree, the structures of the two are basically the same, all showing the two tribes of Typhlocybini and Zyginellini clustered into one clade. However, relationships within Erythroneurini, the position of the clade comprising *M.* *protuberanta* and *Illinigina* sp*.* differs. This may be attributable to the differences in taxon sample between the two analyses, i.e., the absence of Empoascini in the morphology-based phylogeny and the different outgroups used to root the trees.

## Conclusions

The Oriental leafhopper genus *Cassianeura* (Cicadellidae: Typhlocybinae: Erythroneurini) contains three species which feed and reproduce on *Cassia* trees. In this study, we sequenced and annotated the complete mitogenomes of two *Cassianeura* species (*C.* *cassiae* and *C.* *bimaculata*) and compared them with other Typhlocybinae leafhoppers. Comparative mitogenome analysis showed that the gene content and gene arrangement of the two new mitogenomes are both conservative. Phylogenetic analysis of the nucleotide sequences of 13 PCGs resulted in a well-supported topology, with most branches receiving strong support and most relationships consistent with the results of other recent phylogenetic studies. For example, Zhou et al. supports treating Zyginellini as a synonym of Typhlocybini^49^.

Comparative morphological study of the ovipositors of 9 typhlocybine leafhoppers showed that species in the same genus have similar structure, but there are obvious morphological differences among genera and the shape of female sternite VII of different species is quite different. Morphology based phylogenetic analysis of Typhocybinae yields a tree similar to that obtained by analyzing mitogenome sequences. Both suggest that Typhlocybini and Zyginellini are closely related but neither support the monophyly of either tribe. The results of this study provide further comparative data on the mitogenomes of Typhlocybinae and suggest that morphological and mitogenome data provide consistent phylogenetic signal useful for elucidating relationships among genera and tribes of this diverse group of plant-feeding insects.

## Supplementary Information

Supplementary Information.
