# Supplementary material for: Mitogenome and phylogenetic analysis of typhlocybine leafhoppers (Hemiptera: Cicadellidae)
Source: Sci Rep. 2021 May 12;11:10053. doi: 10.1038/s41598-021-89525-5 (PMC8115509; doi:10.1038/s41598-021-89525-5)
Supplement: Supplementary file 1 — Supplementary Information. [file 41598_2021_89525_MOESM1_ESM.doc]

**Mitogenome and phylogenetic analysis of typhlocybine leafhoppers (Hemiptera: Cicadellidae)**

Jia Jiang^1,2^, Xiaoxiao Chen^1,2^, Can Li^2^ & Yuehua Song^1^

^1^School of Karst Science, Guizhou Normal University / State Key Laboratory Cultivation Base for Guizhou Karst Mountain Ecology Environment of China, Guizhou Guiyang 550001, China. ^2^Guizhou Provincial Key Laboratory for Rare Animal and Economic Insect of the Mountainous Region / Guizhou Provincial Engineering Research Center for Biological Resources Protection and Efficient Utilization of the Mountainous Region, Guiyang University, Guiyang, Guizhou 550005, China. Correspondence and requests for materials should be addressed to Y.S. (email: songyuehua@163.com)

**Supplementary Information**

**Table S1.** Organization of the *C. cassiae* and *C. bimaculata* genomes

| **Feature** | **Strand** | ***C. cassiae*** | | | | ***C. bimaculata*** | | | |
| --- | --- | --- | --- | --- | --- | --- | --- | --- | --- |
|  |  | **Position** | **Size(bp)** | **Intergenic nucleotides** | **Start/Stop codon** | **Position** | **Size(bp)** | **Intergenic nucleotides** | **Start/Stop codon** |
| *trnI* | N | 1-65 | 65 |  |  | 1-64 | 64 |  |  |
| *trnQ* | J | 63-131 | 69 | -3 |  | 62-130 | 69 | -3 |  |
| *trnM* | N | 138-206 | 69 | 6 |  | 141-207 | 67 | 10 |  |
| *nad2* | N | 207-1178 | 972 |  | ATC/TAA | 208-1179 | 972 |  | ATT/TAA |
| *trnW* | N | 1177-1242 | 66 | -2 |  | 1178-1243 | 66 | -2 |  |
| *trnC* | J | 1235-1296 | 62 | -8 |  | 1236-1297 | 62 | -8 |  |
| *trnY* | J | 1301-1370 | 70 | 4 |  | 1306-1367 | 62 | 8 |  |
| *cox1* | N | 1369-2904 | 1536 | -2 | ATG/TAA | 1369-2904 | 1536 | 1 | ATG/TAA |
| *trnL2* | N | 2911-2977 | 67 | 6 |  | 2909-2975 | 67 | 4 |  |
| *cox2* | N | 2978-3656 | 679 |  | ATA/T | 2976-3654 | 679 |  | ATC/T |
| *trnK* | N | 3657-3728 | 72 |  |  | 3655-3726 | 72 |  |  |
| *trnD* | N | 3728-3793 | 66 | -1 |  | 3727-3788 | 62 |  |  |
| *atp8* | N | 3792-3944 | 153 | -2 | TTG/TAG | 3787-3939 | 153 | -2 | TTG/TAA |
| *atp6* | N | 3938-4591 | 654 | -7 | ATG/TAA | 3933-4586 | 654 | -7 | ATG/TAA |
| *cox3* | N | 4593-5372 | 780 | 1 | ATG/TAA | 4588-5367 | 780 | 1 | ATG/TAA |
| *trnG* | N | 5372-5435 | 64 | -1 |  | 5369-5430 | 62 | 1 |  |
| *nad3* | N | 5436-5789 | 354 |  | ATT/TAA | 5431-5784 | 354 |  | ATC/TAA |
| *trnA* | N | 5791-5853 | 63 | 1 |  | 5788-5849 | 62 | 3 |  |
| *trnR* | N | 5864-5926 | 63 | 10 |  | 5849-5910 | 62 | -1 |  |
| *trnN* | N | 5925-5991 | 67 | -2 |  | 5910-5973 | 64 | -1 |  |
| *trnS1* | N | 5991-6058 | 68 | -1 |  | 5973-6040 | 68 | -1 |  |
| *trnE* | N | 6064-6128 | 65 | 5 |  | 6040-6106 | 67 | -1 |  |
| *trnF* | J | 6134-6199 | 66 | 5 |  | 6105-6168 | 64 | -2 |  |
| *nad5* | J | 6199-7869 | 1671 | -1 | TTG/TAA | 6169-7837 | 1669 |  | TTG/T |
| *trnH* | J | 7870-7932 | 63 |  |  | 7838-7901 | 64 |  |  |
| *nad4* | J | 7938-9266 | 1329 | 5 | ATG/TAA | 7901-9229 | 1329 | -1 | ATG/TAA |
| *nad4L* | L | 9260-9538 | 279 | -7 | ATG/TAA | 9223-9501 | 279 | -7 | ATG/TAA |
| *trnT* | N | 9541-9607 | 67 | 2 |  | 9510-9573 | 64 | 8 |  |
| *trnP* | J | 9608-9672 | 65 |  |  | 9574-9640 | 67 |  |  |
| *nad6* | N | 9675-10163 | 489 | 2 | ATT/TAA | 9643-10128 | 486 | 2 | ATT/TAA |
| *cytb* | N | 10166-11302 | 1137 | 2 | ATG/TAG | 10132-11268 | 1137 | 3 | ATG/TAG |
| *trnS2* | N | 11301-11364 | 64 | -2 |  | 11267-11332 | 66 | -2 |  |
| *nad1* | J | 11364-12296 | 933 | -1 | ATT/TAA | 11326-12264 | 939 | -7 | ATT/TAA |
| *trnL1* | J | 12297-12362 | 66 |  |  | 12265-12330 | 66 |  |  |
| *rrnL* | J | 12363-13546 | 1184 |  |  | 12331-13498 | 1168 |  |  |
| *trnV* | J | 13547-13612 | 66 |  |  | 13499-13564 | 66 |  |  |
| *rrnS* | J | 13613-14345 | 733 |  |  | 13565-14295 | 731 |  |  |
| *D-loop* |  | 14346-15423 | 1078 |  |  | 14296-14597 | 302 |  |  |

**Table S2.** Codon usage of *C. cassiae* (C) and *C. bimaculata* (B) PCGs

| **Amino** | | **Count/RSCU** | | | | **Amino** | | **Count/RSCU** | | | |
| --- | --- | --- | --- | --- | --- | --- | --- | --- | --- | --- | --- |
| **Acid** | **Codon** | **C** | | **B** | | **Acid** | **Codon** | **C** | | **B** | |
| Phe | UUU | 204 | 1.53 | 231 | 1.59 | Tyr | UAU | 190 | 1.53 | 128 | 1.52 |
|  | UUC | 63 | 0.47 | 59 | 0.41 |  | UAC | 59 | 0.47 | 40 | 0.48 |
| Leu2 | UUA | 299 | 3.93 | 213 | 3.12 | His | CAU | 56 | 1.44 | 59 | 1.53 |
|  | UUG | 43 | 0.56 | 49 | 0.72 |  | CAC | 22 | 0.56 | 18 | 0.47 |
| Leu1 | CUU | 27 | 0.35 | 57 | 0.84 | Gln | CAA | 52 | 1.51 | 76 | 1.54 |
|  | CUC | 17 | 0.22 | 12 | 0.18 |  | CAG | 17 | 0.49 | 23 | 0.46 |
|  | CUA | 55 | 0.72 | 64 | 0.94 | Asn | AAU | 315 | 1.52 | 264 | 1.7 |
|  | CUG | 16 | 0.21 | 14 | 0.21 |  | AAC | 99 | 0.48 | 47 | 0.3 |
| Ile | AUU | 243 | 1.51 | 303 | 1.78 | Lys | AAA | 258 | 1.51 | 245 | 1.67 |
|  | AUC | 78 | 0.49 | 37 | 0.22 |  | AAG | 84 | 0.49 | 49 | 0.33 |
| Met | AUA | 223 | 1.7 | 208 | 1.63 | Asp | GAU | 35 | 1.37 | 57 | 1.73 |
|  | AUG | 40 | 0.3 | 47 | 0.37 |  | GAC | 16 | 0.63 | 9 | 0.27 |
| Val | GUU | 32 | 1.42 | 59 | 1.92 | Glu | GAA | 57 | 1.61 | 96 | 1.6 |
|  | GUC | 8 | 0.36 | 9 | 0.29 |  | GAG | 14 | 0.39 | 24 | 0.4 |
|  | GUA | 42 | 1.87 | 43 | 1.4 | Cys | UGU | 30 | 1.36 | 33 | 1.61 |
|  | GUG | 8 | 0.36 | 12 | 0.39 |  | UGC | 14 | 0.64 | 8 | 0.39 |
| Ser2 | UCU | 46 | 1.26 | 73 | 1.92 | Trp | UGA | 46 | 1.26 | 75 | 1.56 |
|  | UCC | 20 | 0.55 | 22 | 0.58 |  | UGG | 27 | 0.74 | 21 | 0.44 |
|  | UCA | 71 | 1.95 | 65 | 1.71 | Arg | CGU | 8 | 1.14 | 15 | 1.54 |
|  | UCG | 7 | 0.19 | 14 | 0.37 |  | CGC | 3 | 0.43 | 1 | 0.1 |
| Pro | CCU | 26 | 1.68 | 59 | 1.92 |  | CGA | 14 | 2 | 18 | 1.85 |
|  | CCC | 6 | 0.39 | 16 | 0.52 |  | CGG | 3 | 0.43 | 5 | 0.51 |
|  | CCA | 27 | 1.74 | 41 | 1.33 | Ser1 | AGU | 41 | 1.13 | 41 | 1.08 |
|  | CCG | 3 | 0.19 | 7 | 0.23 |  | AGC | 27 | 0.74 | 15 | 0.39 |
| Thr | ACU | 47 | 1.35 | 74 | 1.99 |  | AGA | 43 | 1.18 | 50 | 1.32 |
|  | ACC | 27 | 0.78 | 22 | 0.59 |  | AGG | 36 | 0.99 | 24 | 0.63 |
|  | ACA | 55 | 1.58 | 48 | 1.29 | Gly | GGU | 27 | 1.61 | 50 | 1.87 |
|  | ACG | 10 | 0.29 | 5 | 0.13 |  | GGC | 1 | 0.06 | 8 | 0.3 |
| Ala | GCU | 22 | 2.26 | 30 | 1.56 |  | GGA | 30 | 1.79 | 28 | 1.05 |
|  | GCC | 2 | 0.21 | 14 | 0.73 |  | GGG | 9 | 0.54 | 21 | 0.79 |
|  | GCA | 15 | 1.54 | 29 | 1.51 | * | UAA | 198 | 1.65 | 145 | 1.74 |
|  | GCG | 0 | 0 | 4 | 0.21 |  | UAG | 42 | 0.35 | 22 | 0.26 |

**
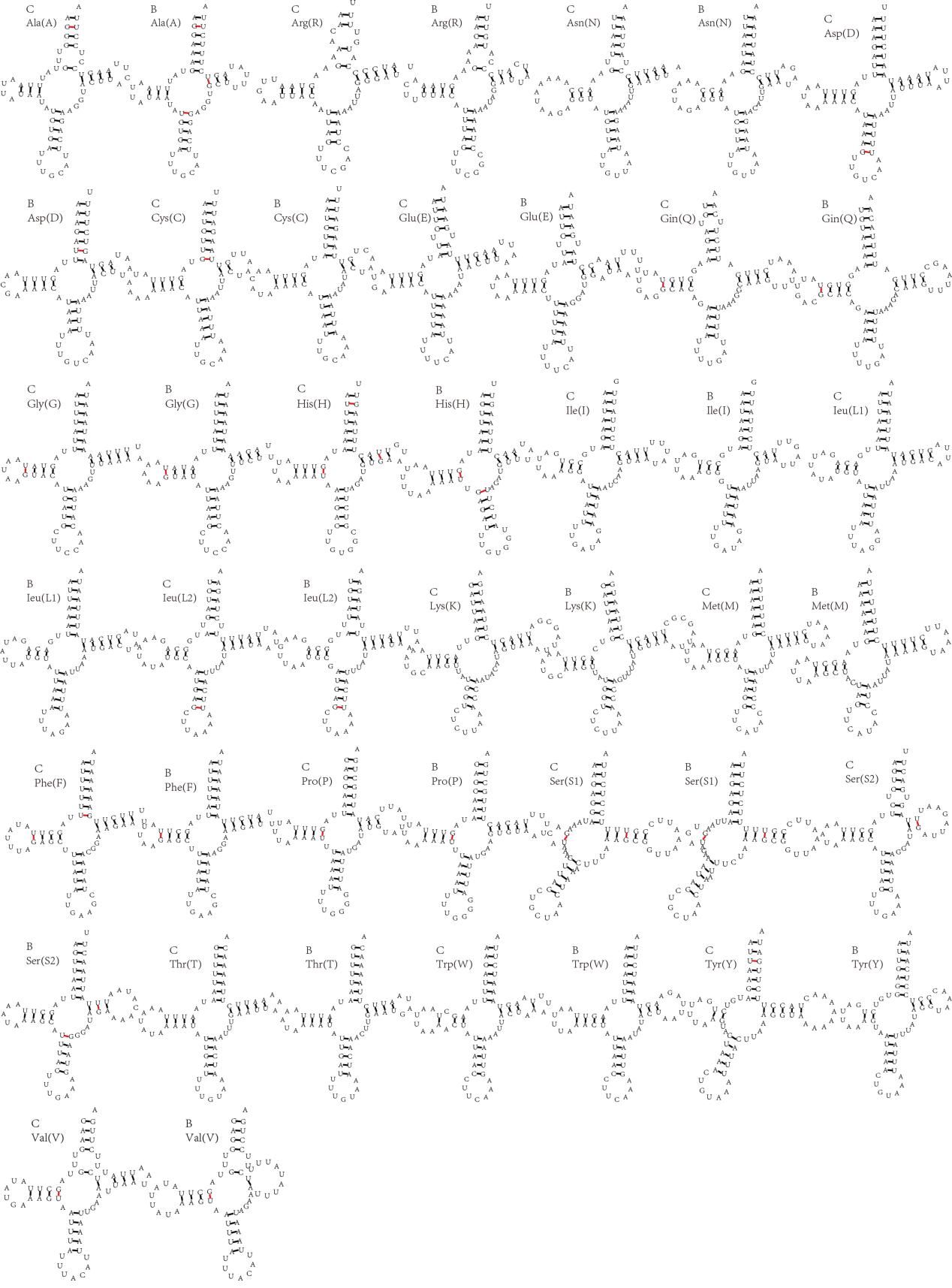
**

**Figure S1.** Inferred secondary structure of 22 tRNAs of *C. cassiae* (C) and *C. bimaculata* (B) mitogenomes. Black lines indicate Watson-Crick base pairing, and red line indicate G-U base pairing.

**
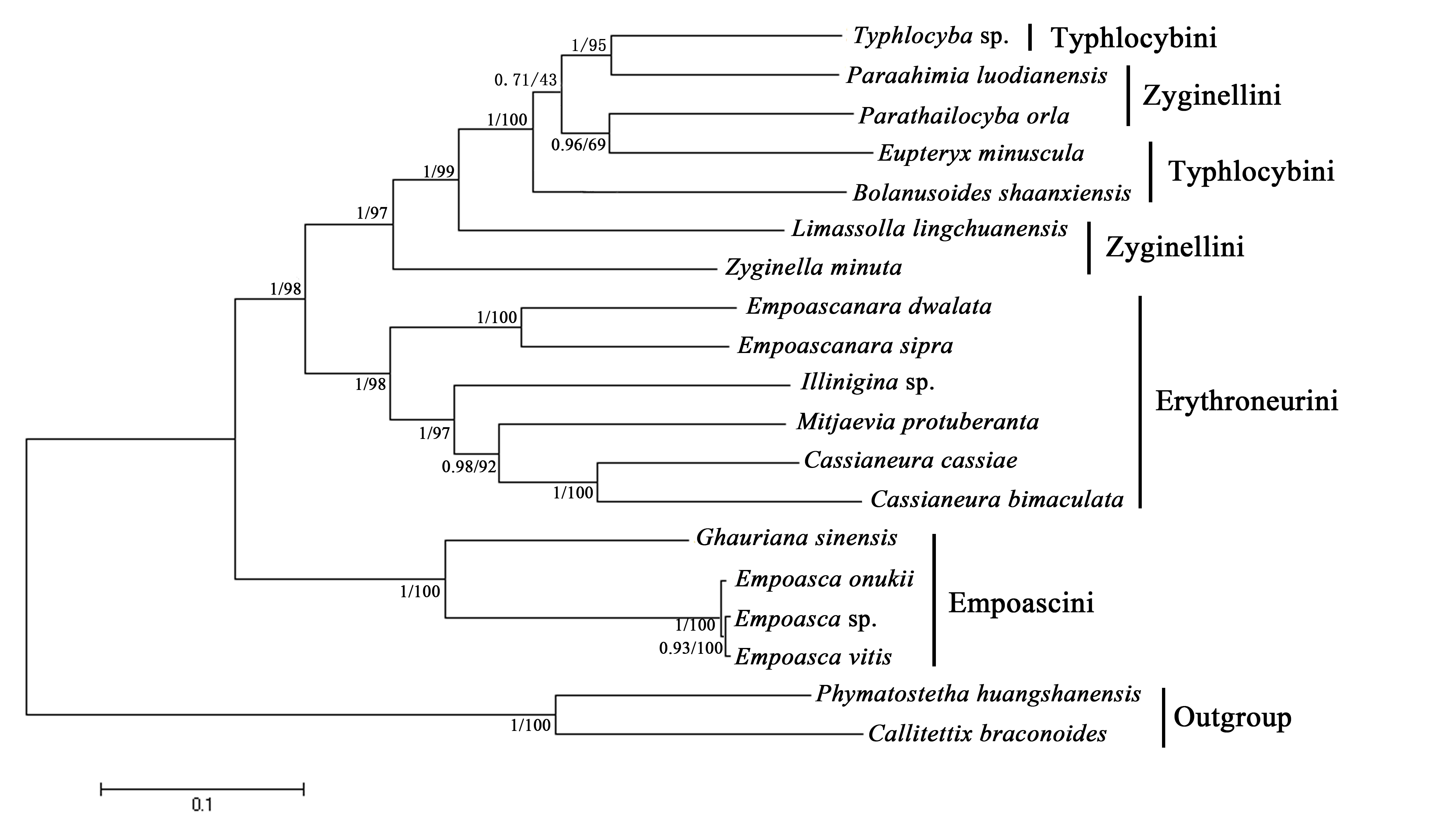
**

**Figure S2.** Phylogenetic tree from Typhlocybinae based on nucleotide sequence of the first and second codons of 13 PCGs. Numbers above the nodes refer to the posterior probability (left) of Bayesian (BI) analyses and bootstrap proportion (right) of maximum likelihood (ML) analyses.

**
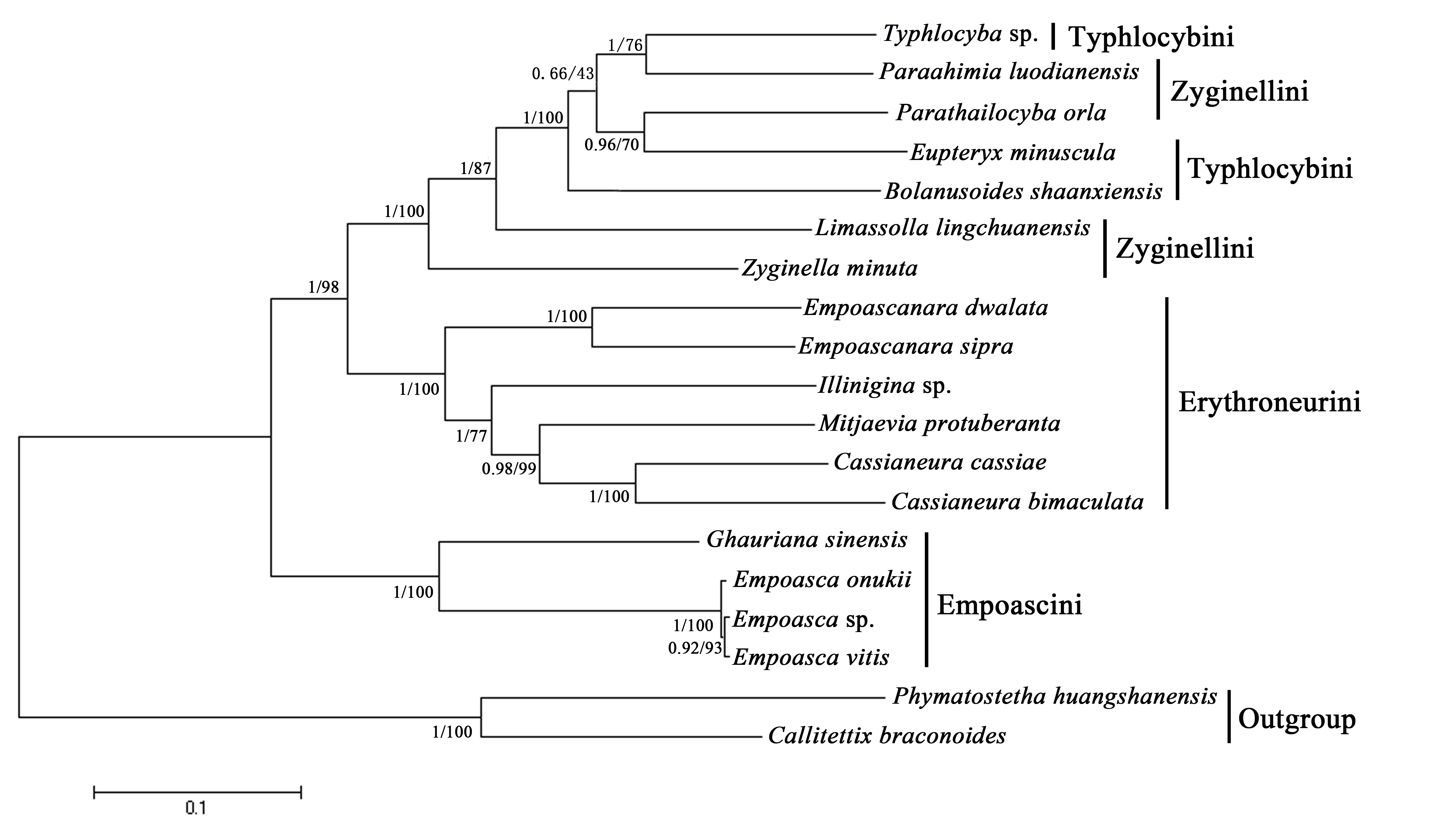
**

**Figure S3.** Phylogenetic tree from Typhlocybinae based on nucleotide sequence of 13 PCGs and two rRNAs. Numbers above the nodes refer to the posterior probability (left) of Bayesian (BI) analyses and bootstrap proportion (right) of maximum likelihood (ML) analyses.

**Table S3.** 63 morphological traits of phylogenetic analysis of Typhlocybinae.

| Number | Traits | Character evolution series |
| --- | --- | --- |
| **Female valvula** | | |
| 1 | Female valvula I | 0, slender; 1, relatively wide |
| 2 | Female valvula I | 0, Suddenly thin; 1, evenly thinner from base to end |
| 3 | The dorsal side of a female valvula Ⅱ | 0, coarsely serration consisting of many small serrations; 1, serrations of varying sizes; 2, large serrations of uniform size; 3, slanted big serration |
| 4 | The ventral preapical of a female valvula Ⅱ | 0, serrated; 1, smooth |
| 5 | The dorsal side of another female valvula Ⅱ | 0, small serration; 1, big serration |
| 6 | The dorsal side of another female valvula Ⅱ | 0, serrations of varying sizes; 1, small serrations of uniform size |
| 7 | The ventral preapical of another female valvula Ⅱ | 0, serrated; 1, smooth |
| 8 | Female valvula Ⅲ | 0, fine and dense bristles; 1, relatively few bristles |
| **Coloration** | | |
| 9 | Ground color of dorsum | 0, pale yellow or white; 1, dark reddish or brown |
| 10 | Vertex markings | 0, unicolorous or with indistinct markings; 1, with pair of dark preapical spots; 2, with large median apical spot; 3, other |
| 11 | Anteclypeus | 0, concolorous with rest of face; 1, darker than other parts, brown or black |
| 12 | Pronotum | 0, without Y- or V-shaped medial vitta; 1, with Y- or V-shaped medial vitta |
| 13 | Scutellum | 0, without dark lateral triangles; 1, with dark lateral triangles |
| 14 | Forewing | 0, without numerous irregular red dots; 1, with numerous irregular red dots |
| 15 | Forewing | 0, without oblique vittae; 1, with oblique vittae |
| 16 | Clavus | 0, unicolorous; 1, with separate basal and distal vittae; 2, with continuous vitta parallel to suture |
| 17 | Costal margin of forewing | 0, without dark spot; 1, with dark spot |
| 18 | Third apical cell | 0, without brown spot; 1, with brown spot |
| 19 | First apical cell | 0, without brown spot; 1, with brown spot |
| **Head** | | |
| 20 | Crown | 0, narrower than pronotum; 1, as wide as or wider than pronotum |
| 21 | Crown | 0, weakly produced, anterior and posterior margins parallel; 1, anterior margin slightly produced; 2, anterior margin strongly produced and angulate medially |
| 22 | Coronal suture | 0, extended to crown aoex; 1, incomplete, not reaching crown apex; 2, absent or very short |
| 23 | Face in profile | 0, not depressed, 45°or more from horizontal; 1, depressed, less than 45°from horizontal |
| **Thorax** | | |
| 24 | Forewing vein CuA | 0, distinctly shorter than CuP; 1, subequal to CuP; 2, distinctly longer than CuP |
| 25 | First apical cell base | 0, oblique, apex of Cu and m-cu crossvein forming continuous line; 1, distinctly angulate; 2, transverse |
| 26 | Third apical cell | 0, quadrate; 1, petiolate |
| 27 | Second apical cell | 0, widened distally; 1, parallel sided; 2, narrowed distally |
| 28 | Second apical cell | 0, straight; 1, curved |
| 29 | Fourth apical cell | 0, short, length < 2 times width; 1, length subequal to 2 times width; 2, elongate, length distinctly greater than 2 times width |
| 30 | Claval vein | 0, visible throughout length; 1, obsolete |
| 31 | Hindwing apex | 0, broadly rounded; 1, distinctly narrowed ; 2, truncate |
| 32 | Hindwing vein RA | 0, present; 1, absent |
| 33 | Hindwing veins MP and CuA | 0, separale, connected by crossvein, or touching at one point: 1, fused for short distance, then divergent; 2, completely confluent |
| 34 | Hindwing veins MP and CuA | 0, divergent distally; 1, parallel; 2, convergent distally; 3, CuA reduced or absent distally |
| **Male pygofer** | | |
| 35 | 2S abdominal apodemes | 0, small, narrow, extended dorsomesad; l, large, broad, extended to or near 3S posterior margin; 2, large, broad, extended beyond 3S posterior margin |
| 36 | Pygofer length relative to subgenital plate | 0, shorter; 1. subequal; 2, longer |
| 37 | Pygofer dorsoapical lobe | 0, rounded; I, angulate; 2. acutely produced |
| 38 | Pygofer dorsal macrosetae | 0, absent; I,one or two present; 2, several present |
| 39 | Pygofer distal setae | 0, undifferentiated; I, in distinct group |
| 40 | Pygofer long fine setae | 0, absent, 1, present, sparse; 2, numerous, conspicuous |
| 41 | Pygofer microtrichia | 0, absent or inconspicuous; 1, well developed, at least distally |
| 42 | Pygofer dorsal appendage | 0, movably articulated, ventral angle of base free; 1, with distinct basal suture, but not movably articulated; 2, fused, without suture; 3, absent |
| 43 | Pygofer dorsal appendage length | 0, not reaching pygofer apex; 1, reaching pygofer apex; 2, extended beyond pygofer apex |
| 44 | Pygofer dorsal appendage, dorsal view | 0, straight or very slightly curved; 1, distinctly sinuate |
| 45 | Pygofer dorsal appendage, lateral view | 0, straight; 1, curved upward; 2, curved downward |
| 46 | Pygofer ventral appendage | 0, present; 1, absent |
| **Subgenital plate** | | |
| 47 | Subgenital plate proportions | 0, section basad of medial constriction subequal to or shorter than distal section; 1, basal section longer than distal section |
| 48 | Subgenital plate basal macrosetae | 0, absent; 1, one; 2, two-four; 3, five-seven; 4, nine or more |
| 49 | Subgenital plate macrosetal row | 0, uniseriate, along margin; 1, oblique, extended posteromesad; 2, scattered; 3, other |
| 50 | Subgenital plate marginal subbasal setae | 0, absent or poorly differentiated; 1, distinct, peglike, forming continuous row; 2, peglike setae restricted to basolateral angle; 3, group of macrosetae present; 4, long fine setae present |
| 51 | Subgenital plate distal macrosetae | 0, absent; 1, present |
| **Style** | | |
| 52 | Style preapical lobe | 0, absent or weak; 1, prominent; 2, cheliform; 3. greatly enlarged; 4, acuminate; 5, auriculate |
| 53 | Style apex | 1, serrate; 2, crenulate |
| 54 | Style apex | 0, slender; 1, truncate and expanded (footlike); 2, with 3 points |
| **Aedeagus** | | |
| 55 | Aedeagus dorsal apodeme, lateral view | 0, absent; 1, present, not expanded in lateral view; 2, broadly expanded in lateral view |
| 56 | Aedeagus dorsal apodeme, ventral view | 0, very short or long and parallel-sided, connected to pygofer membranous; 1, with U- or V-shaped dorsolateral ligaments connected to anal tube and/or pygofer appendage; 2, triangular or T-shaped, without distinct connection to anal tube or pygofer appendage |
| 57 | Aedeagus preatrium | 0, shorter than or equal to stem length; 1, greater than stem length |
| 58 | Aedeagal shaft, ventral view | 0, symmetrical; I, asymmetrical |
| 59 | Aedeagal shaft, lateral view | 0, slender; 1, broad |
| 60 | Aedeagal shaft | 0, smooth; 1, denticulate distally |
| **Connective** | | |
| 61 | Connective, median anterior lobe | 0, absent; 1, present, broad; 2, present, slender |
| 62 | Connective arms | 0, short; 1, long |
| 63 | Connective stem | 0, absent or very short; 1, well developed, entire; 2, bifid |

**Table S4.** Characteristic data matrix for phylogenetic analysis.

| **Species** | **Characteristic state** |
| --- | --- |
| *Mitjaevia protuberanta* Song, Li & Xiong, 2011 | 0100010001 1010000000 0101001010 1011000000 1000201221 0101111010 201 |
| *Cassianeura cassiae* (Ahmed, 1970) | 0121011101 0010000001 0201101020 1111200002 1200200400 0101120000 110 |
| *Cassianeura bimaculata* Dworakowska, 1984 | 0121011101 00?0000001 0201101020 1111200002 1200200203 0100220000 011 |
| *Empoascanara sipra* Dworakowska, 1980 | 0110011012 1010000001 1101101020 1102000000 0000200201 0101121000 201 |
| *Empoascanara dwalata* Dworakowska, 1971 | 0110011012 1010000001 1101101020 1102000000 0000110201 0101120010 201 |
| *Eupteryx minuscula* Lindberg, 1929 | 0131000113 0010000110 2102110101 2121200001 13???11131 0111120110 101 |
| Eupteryx gracilivramus Hou, Zhang & Huang, 2016 | 0131000010 0010000000 2102110100 2121200001 13???11131 0011120000 211 |
| *Limassolla lingchuanensis* Chou & Zhang, 1985 | 0100011103 0111111110 2112110101 1123221000 13???11201 0001120010 002 |
| *Zyginella minuta* (Yang, 1965) | 1001111013 000121110 2112110101 2123200210 13???11300 0000120000 011 |
| *Parathailocyba oral* (Dworakowska, 1977) | ????????10 0010000110 2112110101 2121200000 13???01130 0000120100 101 |
| *Paraahimia luodianensis* Yuan & Song, 2019 | ????????10 0010011111 2100110101 1121200000 13???01131 0000100000 001 |
| *Typhlocyba* sp. | ????????13 ?010011111 21?0110101 1???201002 13???0?130 0000100000 001 |
| *Bolanusoides shaanxiensis* Huang & Zhang, 2005 | ????????00 0010011110 2???100101 1021??0212 13???0?13? 0000100010 001 |
| *Illinigina* sp. | ????????02 ?010000000 0101001010 1111000002 1200201201 0101210010 010 |
| *Dikraneura* sp. | ????????00 0000000001 2010201020 100?20?200 1000000411 1200200010 010 |
